# Hypermethylation of Mest promoter causes aberrant Wnt signaling in patients with Alzheimer’s disease

**DOI:** 10.1038/s41598-021-99562-9

**Published:** 2021-10-08

**Authors:** Renuka Prasad, Hwajin Jung, Anderson Tan, Yonghee Song, Sungho Moon, Mohammed R. Shaker, Woong Sun, Junghee Lee, Hoon Ryu, Hyun Kook Lim, Eek-hoon Jho

**Affiliations:** 1 Department of Life Science, University of Seoul, Seoul, 02504 Republic of Korea; 2 Department of Anatomy, Korea University College of Medicine, Seoul, 02841 Republic of Korea; 3 Boston University Alzheimer’s Disease Center and Department of Neurology, Boston University School of Medicine, Boston, MA 02118 USA; 4 Center for Neuroscience, Brain Science Institute, Korea Institute of Science and Technology, Seoul, 02792 Republic of Korea; 5 Department of Psychiatry, Yeouido St. Mary’s Hospital, College of Medicine, The Catholic University of Korea, Seoul, Republic of Korea

**Keywords:** Neuroscience, Cell death in the nervous system

## Abstract

Alzheimer's disease (AD) is a progressive neurodegenerative disorder that leads to dementia and behavioral changes. Extracellular deposition of amyloid plaques (Aβ) and intracellular deposition of neurofibrillary tangles in neurons are the major pathogenicities of AD. However, drugs targeting these therapeutic targets are not effective. Therefore, novel targets for the treatment of AD urgently need to be identified. Expression of the mesoderm-specific transcript (Mest) is regulated by genomic imprinting, where only the paternal allele is active for transcription. We identified hypermethylation on the Mest promoter, which led to a reduction in Mest mRNA levels and activation of Wnt signaling in brain tissues of AD patients. Mest knockout (KO) using the CRIPSR/Cas9 system in mouse embryonic stem cells and P19 embryonic carcinoma cells leads to neuronal differentiation arrest. Depletion of Mest in primary hippocampal neurons via lentivirus expressing shMest or inducible KO system causes neurodegeneration. Notably, depletion of Mest in primary cortical neurons of rats leads to tau phosphorylation at the S199 and T231 sites. Overall, our data suggest that hypermethylation of the Mest promoter may cause or facilitate the progression of AD.

## Introduction

Misregulation of Wnt/β-catenin signaling is one of the major causes of diseases, due to the role of this pathway in cell proliferation, self-renewal, and cell fate determination during embryonic development and tissue homeostasis in adults^1^. Mutations in Wnt signaling components are tightly linked with age-related neurodegenerative conditions, including Alzheimer's disease (AD)^2^. Previous studies have reported an association between single nucleotide polymorphisms in LRP6 (LDLR (low-density lipoprotein receptor)-related protein 6), a co-receptor of Wnt ligand, and late-onset AD. The LRP6 Val 1062 allele showed reduced β-catenin signaling^3^. Mesoderm-specific transcript (Mest) is a paternally expressed gene that is abundantly expressed in the mesodermal tissue and adult brain^4,5^. Mest belongs to the α-β hydrolase protein family^6^. Although several reports have shown that Mest regulates diverse biological processes, its exact biochemical function has not yet been characterized. We previously showed that Mest acts as a negative regulator of the Wnt/β-catenin pathway by inhibiting the maturation and membrane localization of LRP6^7^. In addition, Mest knockdown blocked adipocyte differentiation via Wnt signaling activation. Mest also plays a role in brain development by regulating the neuronal migration required for the formation of the neocortex^8^. Furthermore, Mest is specifically expressed in the substantia nigra (SN) and is required for the maintenance of SN neuronal subset^9^. Neuronal loss due to Mest ablation is likely due to the disruption in Wnt signaling. Recently, Mest has also been reported to be involved in the maturation of mammary glands^10^.

Genomic imprinting refers to the mono-allelic expression of a specific gene due to parent-specific methylation of the gene’s promoter^11^. Mest is expressed only from the paternal genome, since the maternal gene promoter is methylated. Loss of imprinting (LOI) of Mest is often correlated with altered growth^12^. Mest is a candidate gene for Silver-Russell Syndrome^13^, and hypermethylation of the Mest promoter is associated with oligozoospermia^14^. In addition, LOI of Mest is also involved in breast, colorectal, and lung cancers^15–17^. Genomic imprinting is loosely controlled during aging. Since aberrant Mest hypermethylation and LOI are often associated with various diseases, it is vital to study the functional role and methylation status of the Mest promoter in AD patients.

Epigenome-wide association studies in AD patients suggested that the Mest promoter is potentially hypermethylated in the cortex of AD patients^18^. However, the underlying mechanism of how Mest promoter hypermethylation is associated with AD pathology remains elusive. In this study, we investigated the methylation status of the Mest promoter and its effect on Wnt signaling using neurons and brain samples from AD patients. We showed that the Mest promoter was hypermethylated in AD patients and Mest mRNA levels were reduced, while the Wnt target genes were elevated. Mest depletion resulted in the upregulation of Wnt signaling and tau phosphorylation, causing neurodegeneration.

## Materials and methods

### Postmortem brain samples

Frozen postmortem human cortex samples from nine AD patients and nine age-matched controls were used in this study. Neuropathological examination of age-matched controls and AD human brain samples was performed using procedures previously established at the Boston University Alzheimer’s Disease Center (BUADC). Next of kin provided informed consent for participation and brain donation. Institutional review board approval for ethical permission was obtained through BUADC. The study was reviewed by the Institutional Review Board of the Boston University School of Medicine (Protocol H-28974) and was approved for exemption because it only included tissues collected from post-mortem subjects not classified as human subjects. The study was performed in accordance with the institutional regulatory guidelines and principles of human subject protection in the Declaration of Helsinki. Sample information is listed in Supplementary Tables 1 and 2.

### DNA extraction and bisulfite conversion

Total genomic DNA was isolated from postmortem frontal cortex of AD patients using an AccuPrep® Genomic DNA Extraction Kit (Bioneer, K-3032), according to the manufacturer’s instructions. Each 500 ng of genomic DNA was subjected to bisulfite conversion using an EZ DNA methylation kit (Zymo Research, D5001), according to the manufacturer's instructions.

### Mest promoter amplification and bisulfite sequencing

Individual genomic DNA was amplified by PCR using the primers listed in Supplementary Table 3. Amplification was performed under the following conditions: 95 °C for 10 min, followed by 40 cycles of 95 °C for 30 s, 55 °C for 35 s, and 72 °C for 40 s, and a final extension at 72 °C for 7 min. The PCR products were electrophoresed on a 1% agarose gel and then isolated from the gel slices using a Gel Extraction Kit (ELPIS BIOTECH, EBD-1005, South Korea). The PCR products were subcloned into pGEM-T easy vector (Promega, A1360) and the selected plasmid DNA was extracted using a DNA isolation kit (LaboPass, CMP0112). The plasmid was sequenced using the M13 forward and reverse primers (Bionics, Seoul, South Korea). Sequences were analyzed using BLAST (http://blast.ncbi.nlm.nih.gov).

### Cell culture and transfection

HEK293FT (Invitrogen) cells were maintained in high glucose Dulbecco’s Modified Eagle Medium (DMEM) containing 10% fetal bovine serum (FBS), 0.1 mM MEM non-essential amino acids (NEAA), 2 mM ʟ-glutamine, 1% penicillin/streptomycin, and 500 µg/ml Geneticin, and incubated at 37 °C with 5% CO_2_.

Mouse embryonic stem cells (mESCs) and E14 cell lines were used for the experiments. mESCs were cultured on a tissue culture dish pre-coated with 0.2% gelatin (Sigma) at 37 °C in a 5% CO_2_ incubator. mESC culture medium consisted of Glasgow Minimum Essential Medium (Sigma), 15% fetal bovine serum (Bio West), Tylosine (Sigma), 0.1 mM β-mercaptoethanol (Gibco), 100X nonessential amino acid (Corning), 1 mM sodium pyruvate (Gibco), 100X GlutaMAX (Gibco) and Leukemia Inhibitory Factor (LIF)-conditioned medium. LIF-conditioned medium was prepared as previously described^19^. All cell lines were cultured at 37 °C in a 5% CO_2_ incubator and passaged every 2–3 days.

For neuronal differentiation, 1.0 × 10^4^ undifferentiated mESCs were plated in each well of a 6-well plate pre-coated with 0.2% gelatin. After 24 h, the medium was replaced with N2B27 medium composed of equal amounts of DMEM/F-12 (Welgene, South Korea) and neurobasal media (Gibco) supplemented with N2 (Gibco) medium, B27 (Gibco), GlutaMAX (Gibco), 0.05 mM β-mercaptoethanol (Gibco), and 7.5% BSA (Gibco). The N2B27 medium was changed every 2 days.

For embryonic body (EB) differentiation, mESCs were trypsinized and plated on low-attachment Petri dishes (1000 cells per drop) using the hanging drop technique in mESC culture medium without LIF. After 3 days, EBs were formed and moved to a new tissue culture dish containing fresh mESC culture medium and allowed to differentiate further. The culture medium was replaced every 2 days and cells were harvested at day 0 and day 5.

### P19 cell culture and differentiation

P19 embryonal carcinoma (EC) cells were cultured in minimum essential medium α (Gibco) supplemented with 7.5% newborn calf serum (Gibco), 2.5% fetal bovine serum (Gibco), 100 units/ml penicillin, and 100 μg/ml streptomycin in humidified 5% CO_2_. To induce P19 EC neuronal differentiation, they were aggregated in bacterial-grade Petri dishes containing 1 μM all-trans-retinoic acid (Sigma) at a density of 1 × 10^5^/ml. The medium was replaced with fresh medium containing retinoic acid after 2 days. After 4 days, aggregates were dissociated using 0.05% trypsin, re-plated in poly-D-lysine(PDL) and laminin-coated tissue culture dishes, and then allowed to differentiate in neurobasal medium containing 2% B27 supplement, 0.5 mM ʟ-glutamine, 25 μM ʟ-glutamate, and 1% antibiotics mixture.

### Primary hippocampal culture

Primary mouse hippocampal neuronal cell cultures were prepared from ICR (Young Bio, South Korea) mice, as previously described^20^. Experiments were conducted following standard ethical guidelines and were approved by the University of Seoul Institutional Animal Care and Use Committee (UOS IACUC). Briefly, the hippocampus was isolated from embryonic day 18.5 mouse embryos using Hank’s balanced salt solution. Isolated hippocampi were trypsinized and triturated with fire-polished Pasteur pipettes and then strained through a 40-μm nylon mesh. These cells were then cultured in neurobasal medium containing 2% B27 supplement, 25 μM ʟ-Glutamate, 0.5 mM ʟ-glutamine, and 1% antibiotics mixture. The same growth medium, but without glutamate and antibiotics, was used as replacement medium after 4 days. Half of the medium was changed once a week.

### Lentivirus preparation and infection

Lentiviral short hairpin RNA (shRNA) targeting GFP and shRNA targeting Mest was inserted into a lentivirus vector (Invitrogen, BLOCK-iT™ Lentiviral RNAi Expression System, #K4944-00). The shRNA sequences are listed in Supplementary Table 4. The shRNA lentivirus was packaged in 293FT cells following the manufacturer’s instructions. Briefly, 24 h prior to transfection, ~ 5 × 10^6^ HEK293FT cells were seeded into 100-mm tissue culture plates in DMEM high glucose medium. Transfections were performed using Lipofectamine™ 2000 reagent (Invitrogen). The virus-containing medium was centrifuged at 3,000 rpm for 5 min at 4 °C to remove cell debris and passed through a sterile 0.45-µm filter. The viral supernatant was mixed with PEG-it™ Virus Precipitation Solution (SBI System Biosciences, # LV810A-1) and incubated overnight at 4 °C. After overnight incubation, the mixture was centrifuged, and the viral pellet was resuspended in phosphate-buffered saline (PBS) and titrated using the qPCR Lentivirus Titration Kit (ABM, #LV900). Virus aliquots were stored at -80 °C. The enriched lentivirus particles were used for primary hippocampal neuron infection in the presence of 5 μg/ml polybrene (Sigma).

### Generation of P19 Mest-inducible KO cells using lentiviral split Cas9 system

The lentiviral split Cas9 (LSC-5) vector was digested using the *BsmBI* enzyme. The annealed Mest gRNAs were then ligated into a linearized vector using the golden gate reaction^21^. Lentiviruses for LSC-5 vectors were prepared as previously described^21^. P19 cells were transduced with LSC-5 lentivirus in medium supplemented with 5 μg/mL polybrene (Sigma, #H9268). The transduced cells were selected after 7 days using 3 μg/mL puromycin (Sigma, # P7255). The expression of the LSC-5 vector component FKBP rapamycin binding (FRB) domains was validated in P19 LSC-5 control and Mest gRNA stable cells using qPCR. The stably-selected LSC-5 cells were cultured in rapamycin (200 nM)-containing medium for 5 days. P19 LSC-5 stable cells were incubated in a Petri dish containing retinoic acid and rapamycin for 4 days to induce embryonic body formation. The aggregates were then dissociated into single cells using 0.05% trypsin. Single cells were plated on a PDL-laminin-coated culture dish in neurobasal medium containing B27 and rapamycin for 11 days to promote neuronal differentiation. The Rapamycin-containing medium was changed every day to induce gRNA activation.

### Generation of Mest gene KO lines from P19 and E14 cells using CRISPR-Cas9

The gRNA was designed to target the unique sequences present in Exon 3 of the Mest gene using DNA 2.0 (https://www.atum.bio/). The Mest gRNA cassette was then PCR-amplified from the template plasmid (pLKO-gRNA-Gsk3α) using the primer sequences listed in Supplementary Table 4. The pLKO.1-TRC plasmid was digested with *AgeI* and *EcoRI* to obtain vector fragments. The pLKO.1-Mest gRNA vector was constructed using the NEB Gibson assembly kit (# E5510). For stable expression of Cas9, E14 and P19 cells were co-transfected with PB-CAG-hCas9-IRES-hygro and PiggyBac Transposase vector using Lipofectamine 2000 (Invitrogen), according to the manufacturer’s instructions. The Cas9 stably-expressing cell lines, E14-Cas9 and P19-Cas9, were established by adding hygromycin (200 μg/ml) to the medium for 2 weeks after transfection.

To package the Mest gRNA lentivirus, packaging plasmids psPAX2, VSV-G, and pLKO.1-Mest gRNA vectors were co-transfected into HEK293FT cells using Lipofectamine 2000. After 72 h, the supernatant was collected, passed through 0.45 μm filters (Corning, #431225), and concentrated with PEG-it Virus Precipitation Solution (SBI System Biosciences, # LV810A-1). The concentrated lentivirus was added into the culture medium containing E14 Cas9 and P19 Cas9 stable cells in the presence of 5 μg/ml polybrene (Sigma) to facilitate gRNA expression. After 3 days, puromycin (3 μg/ml) was added to the cell culture medium for 14 days to stably select cells expressing Mest gRNAs.

Genomic DNA was isolated using the AccuPrep Genomic DNA Extraction Kit (Bioneer, Daejon, South Korea) according to the manufacturer’s protocol. PCR amplicons, including Cas9 nuclease target sites, were amplified using the primers listed in Supplementary Table 4. The T7E1 assay was performed as previously described^22^.

### Immunofluorescence analysis

The P19 and primary hippocampal neurons were seeded onto poly-D-lysine and laminin-coated glass coverslips in 12‐well plates, fixed for 10 min in 4% paraformaldehyde in PBS, and then permeabilized at room temperature for 20 min with 0.1% Triton X‐100 in PBS. Coverslips were washed once with PBS and blocked for 2 h with 5% (w/v) bovine serum albumin (Bio basic) in PBS, and then incubated overnight with the primary antibodies. The anti-neuron-specific β-III tubulin (R&D Systems, #MAB1195) antibody and anti-cleaved caspase-3 (Asp175) (Cell Signaling Technology, #9661) antibody was diluted in 1% (w/v) BSA and incubated at 4 °C. The coverslips were then washed three times with PBS and incubated with the secondary antibodies diluted in 1% (w/v) BSA for 2 h. The following secondary antibodies from Invitrogen were used for staining: Alexa Fluor 488 goat anti-rabbit IgG (# A-11034), Alexa Fluor 546 goat anti-mouse IgG (# A-11030), Alexa Fluor 488 goat anti-mouse IgG (# A-11029), and Alexa Fluor 546 goat anti-rabbit IgG (# A-11035). For nuclei staining, Hoechst 33342 solution or DAPI was added to each sample and incubated for 10 min at room temperature. Fluorescent images were captured using a confocal microscope (Carl Zeiss LSM 700).

### Immunoblotting

Cell lysates were prepared in RIPA buffer containing protease inhibitor cocktail tablets (REF 11836170001) and p-stop (REF 04906837001). Lysates were centrifuged at 13,000 rpm for 10 min at 4 °C to isolate soluble proteins. Equal amounts of proteins were boiled with loading sample buffer (NuPAGE LDS; Invitrogen, CA, USA) containing 5% 2-mercaptoethanol for 10 min, resolved using 10% SDS–PAGE, and transferred to a PVDF membrane (Pall Corporation, New York, United States). After blocking with 5% dry milk for 1 h at room temperature, the membranes were incubated overnight at 4 °C with primary antibodies diluted in 5% BSA. The following day, the membranes were washed three times with Tris-buffered saline solution containing 0.1% Tween and then incubated with a horseradish peroxidase-coupled secondary antibody (goat anti-mouse #115-035-003 and goat anti-rabbit #111–035-003, Jackson Immunoresearch Laboratories, PA, USA) or Rabbit TrueBlot antibody (eBioscience, San Diego, USA).

Rabbit monoclonal anti-Mest (#ab151564), anti-Tau (phospho S199) (#ab4749), and anti-Tau (phospho T231) (#ab151559) antibodies were obtained from Abcam. Mouse monoclonal anti-vinculin (#V9131) and anti‐β‐actin antibodies were obtained from Sigma. Rabbit monoclonal anti-LRP6 (#3395S), anti-p-LRP6 (#2568S) antibodies were obtained from Cell Signaling. Mouse monoclonal anti-β-catenin (#610154) antibody was obtained from BD Bioscience and mouse monoclonal anti-active β-catenin (#05-665) antibody was obtained from Millipore. Mouse monoclonal anti-total Tau (#SC-32274) antibody was obtained from Santa Cruz.

### Isolation of mRNA and qPCR analysis

For quantitative real-time PCR, total RNA was isolated using TRIzol reagent (Invitrogen) and cDNA synthesis was performed using ImProm‐II™ Reverse Transcriptase (#A3802, Promega) according to the manufacturer's protocol. Quantitative PCR (qPCR) was carried out using CFX Connect Real-Time PCR (Bio-Rad, Hercules, USA) with SYBR Green PCR Master Mix (Toyobo, Osaka, Japan). The primer sequences used for qPCR are listed in Supplementary Table 3.

### Statistical analysis

All statistical data are expressed as the mean ± SD, and the sample size is indicated in each figure legend. Most experiments were conducted in triplicate. The statistical significance of differences between groups was analyzed using Student’s t-test. *, *p* < 0.05; **, *p* < 0.01; ***, *p* < 0.001 ****, and *p* < 0.0001 represent different degrees of significance.

### Ethics approval and consent to participate

Neuropathological examination of age-matched controls and human brain samples from AD patients was performed using procedures previously established at the Boston University Alzheimer’s Disease Center (BUADC). Next of kin provided informed consent for participation and brain donation. Institutional review board approval for ethical permission was obtained through BUADC. This study was reviewed by the Institutional Review Board of the Boston University School of Medicine (Protocol H-28974) and was approved for exemption because it only included tissues collected from post-mortem subjects not classified as human subjects. The study was performed with informed consent according to protocols approved by the Institutional Review Board of the University of Seoul.

## Results

### Hypermethylation of Mest promoter and upregulation of Wnt signaling in brain samples of AD patients

**Figure 1:**
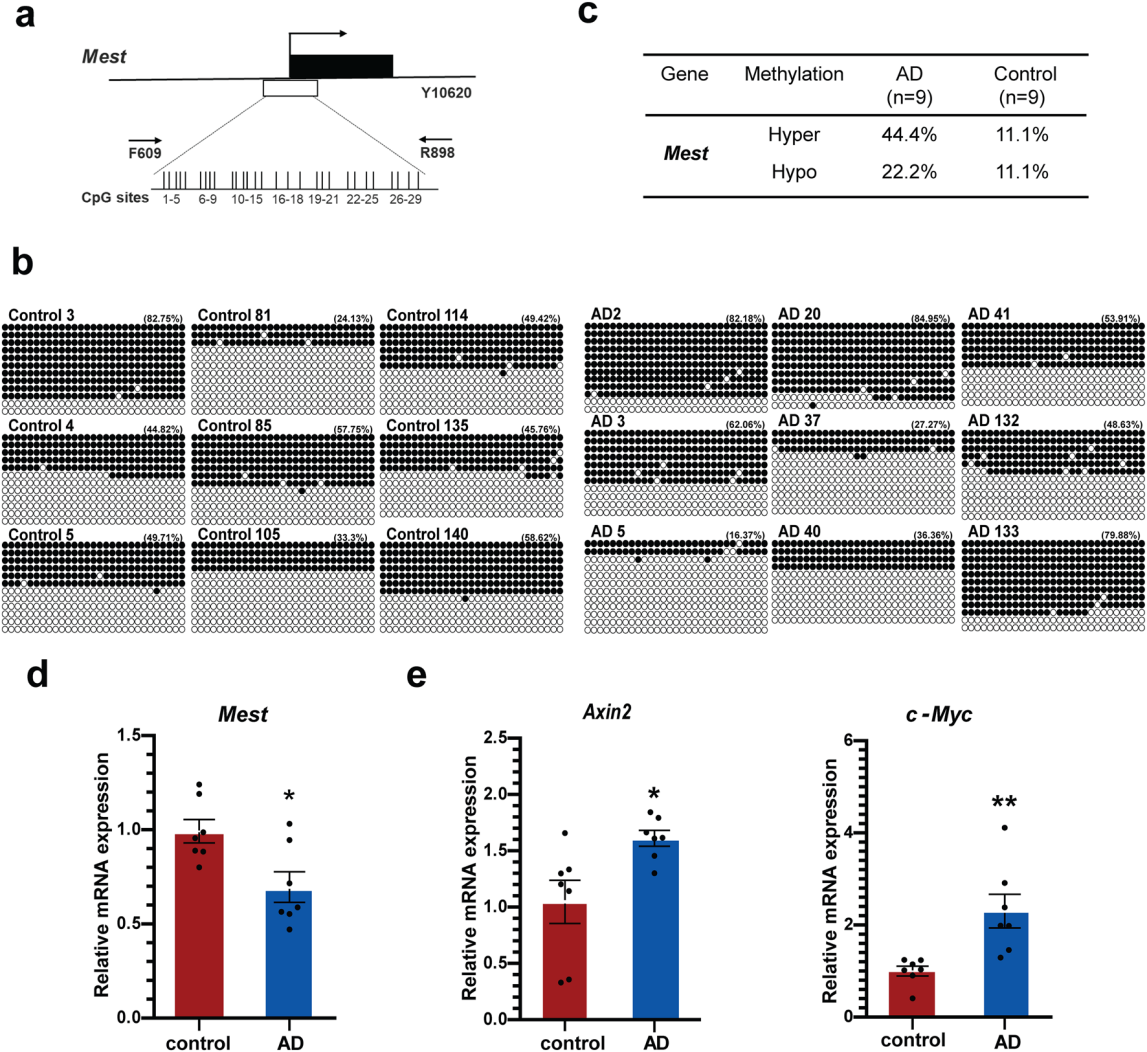
Hypermethylation of Mest promoter and upregulation of Wnt signaling in brain samples from AD patients. (**a**) Schematic diagram of differentially methylated regions (DMRs) of Mest gene promoter (GenBank accession no. Y10620, nucleotides 609–898) is represented as a horizontal line. The transcriptional start site is indicated by an arrow. The vertical bars represent CpG sites. Horizontal arrows (F609 & R898) represent the primers used to amplify the DMRs. (**b**) Comparison of CpG methylation patterns within the Mest promoter in DNA isolated from the cortex of age-matched controls (n = 9) and AD subjects (n = 9) show elevated hypermethylation alleles in AD subjects. (**c**) Summary of Mest promoter methylation in age-matched controls (n = 9) and AD (n = 9) subjects. (**d**) The Mest mRNA levels are decreased in AD patients. Mest mRNA expression is downregulated in the temporal cortex of sporadic AD postmortem brains (n = 7) in comparison to age-matched controls (n = 7). GAPDH was used to normalize Mest expression. *Significantly different from the control at *p* < 0.01. (**e**) mRNA levels of the Wnt target genes Axin2 and c-Myc were increased in AD subjects (n = 7). mRNA levels were normalized to that of β-actin. Data are shown as mean ± SD; *, *p* < 0.05; **, *p* < 0.01; ***, *p* < 0.001 compared with the corresponding control group. Student’s *t* test was used for statistical analysis.

Rao et al*.* reported that the frontal cortex of AD patients showed hypermethylation of CpG islands in brain-derived neurotrophic factor (BDNF), synaptophysin promoters, and increased global DNA methylation^23^. To check whether sporadic AD patients show any differences in CpG methylation in the Mest promoter regions, we analyzed DNA samples extracted from the temporal cortex of postmortem sporadic AD patients and age-matched controls. The clinical characteristics of the control and AD patients are shown in Supplementary Tables 1 and 2. The differentially methylated regions (DMRs) of the Mest gene promoter are shown in Fig. 1a. Interestingly, when hypermethylation and hypomethylation were defined as overall percentage of methylated CpGs of more than 60% or less than 30% in each sample, respectively, 44.4% of the AD cases showed hypermethylation in the Mest promoter in comparison to 11.1% of the age-matched controls (Fig. 1b, c). Our results are consistent with reports that cortex-specific Mest promoter hypermethylation is associated with AD neuropathology^18^.

Mest mRNA levels were decreased in the temporal cortex of sporadic AD postmortem brains in comparison to age-matched controls (n = 7) (Fig. 1d). Canonical Wnt target genes (Axin2 and c-Myc) were also upregulated in AD patients, which is consistent with the previously reported role of Mest as a negative regulator of Wnt signaling (Fig. 1e) ^7^. These results suggest a potential link between hypermethylation of the Mest promoter and elevation of Wnt signaling in AD.

### Mest KO in embryonic carcinoma and embryonic stem cells leads to neuron differentiation blockade

**Figure 2:**
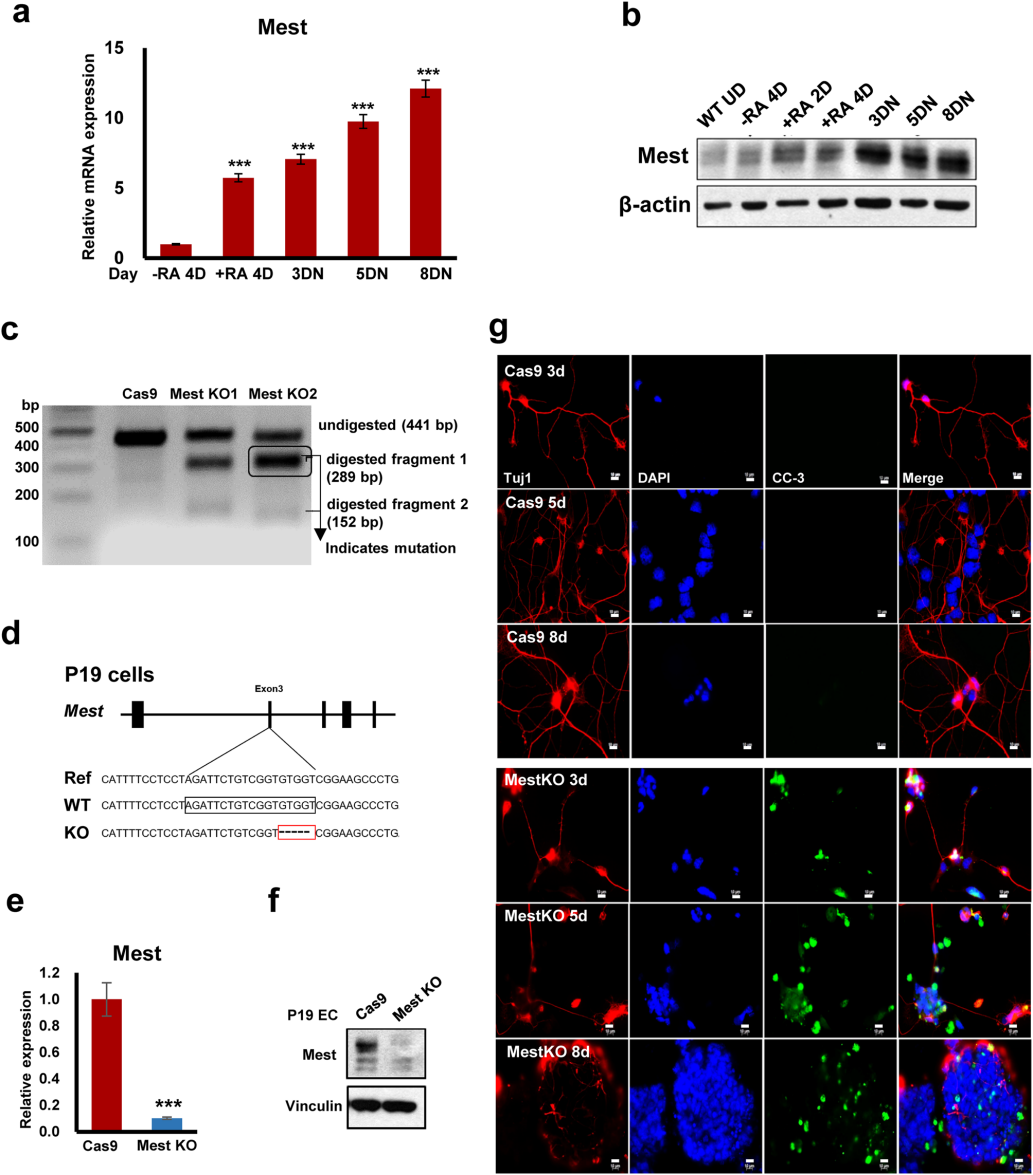
Mest knockout in P19 embryonic carcinoma leads to neuron differentiation blockade. (**a**) qPCR analyses of Mest mRNA expression during neuronal differentiation. Total RNA was isolated at the indicated time points. (**b**) Mest protein levels in retinoic acid induced-P19 neuronal differentiation. (**c**) T7E1 assay for P19 Mest knockout (KO) cells. PCR products were generated from the genomic DNA of P19 Mest KO cells. The PCR products were annealed & digested with T7 Endonuclease I. Fragments were analyzed to determine the efficiency of genome targeting. (**d**) Sanger sequencing of the P19 Mest KO cells showed a 5-base pair deletion near the gRNA target site. The black box indicates the Mest gRNA target region. (**e**) qPCR analyses of Mest mRNA expression in P19 Mest KO cells. (**f**) Mest protein levels in P19 Cas9 and Mest KO cells. (**g**) Mest KO caused neuron differentiation blockade in P19 cells. P19 differentiated neurons were fixed and immunostained with anti-Tuj1 (red) and cleaved caspase-3 ((CC-3), (green)) antibodies at the indicated times. Scale bars, 10 μm. mRNA levels were normalized to that of β-actin. Data are shown as mean ± SD; ***, *p* < 0.001, compared with the corresponding control group. Student’s t test was used for statistical analysis.

To assess the functional significance of Mest in neurons, P19 embryonic carcinoma cells were differentiated into neuronal lineages^24,25^ and the mRNA and protein levels were examined (Supplementary Fig. 1a, b). The levels of both Mest mRNA and protein increased during neuronal differentiation (Fig. 2a, b). Next, we generated P19 Mest KO cells using the CRISPR/Cas9 system. The presence of insertion–deletion (indel) mutations was confirmed using a T7 endonuclease 1 (T7E1) assay and genomic DNA sequencing (Fig. 2c, d). Mest mRNA and protein levels were significantly reduced in KO clones (Fig. 2e, f). P19 Mest KO cells exhibited arrest of neuron differentiation with increased expression of cleaved caspase-3 (Fig. 2g).

We also investigated the role of Mest in the early differentiation of mESCs by generating Mest KO mESCs. Mest protein and mRNA levels were significantly reduced in Mest KO cells compared with the controls (Supplementary Fig. 2a, b). Indel mutations were also observed in Mest KO clones (Supplementary Fig. 2c). Mest levels increased during neuronal differentiation, whereas levels of the neuronal differentiation marker, Nestin, decreased significantly in Mest KO cells (Supplementary Fig. 2d, e). Using the embryonic body (EB) differentiation method, we checked Mest levels during differentiation. We found that Mest expression was downregulated in mESCs and increased with differentiation (Supplementary Fig. 2f). Various lineage-specific markers (Nestin, GSC, and GATA4) were significantly impaired during the differentiation of Mest KO cells, suggesting that Mest depletion affects the differentiation potential of mESCs (Supplementary Fig. 2g–i).

### Mest depletion results in elevation of Wnt signaling and tau phosphorylation, leading to neurodegeneration

To examine the effect of abnormal Wnt/β-catenin signaling levels on neurodegeneration, primary hippocampal neurons of days in vitro (DIV) 7 were treated with small molecules that regulate Wnt signaling. XAV939 antagonizes Wnt signaling by stimulating β-catenin degradation and stabilizing Axin^26^. Hippocampal neurons treated with XAV939 showed increased apoptosis and neurodegeneration (Supplementary Fig. 3a, b, a’). These results are consistent with our previous reports that reduced Wnt signaling leads to neurodegeneration via the small molecule ICG-001^20^. The glycogen synthase kinase (GSK-3) inhibitor, LiCl, was used as a Wnt signaling activator^27^. Treatment with LiCl also resulted in an increase in apoptotic cells and neurodegeneration (Supplementary Fig. 3c, d, b^’^). Similarly, treatment with either XAV939 or LiCl significantly reduced neuronal markers in P19 day 8 neurons (Supplementary Fig. 3e, f). Consistently, Wnt target genes were downregulated and upregulated in the XAV939-and LiCl-treated groups, respectively. Overall, both activation and inhibition of Wnt signaling led to neurodegeneration, indicating that Wnt signaling must be maintained at controlled levels for neurons to survive.

**Figure 3:**
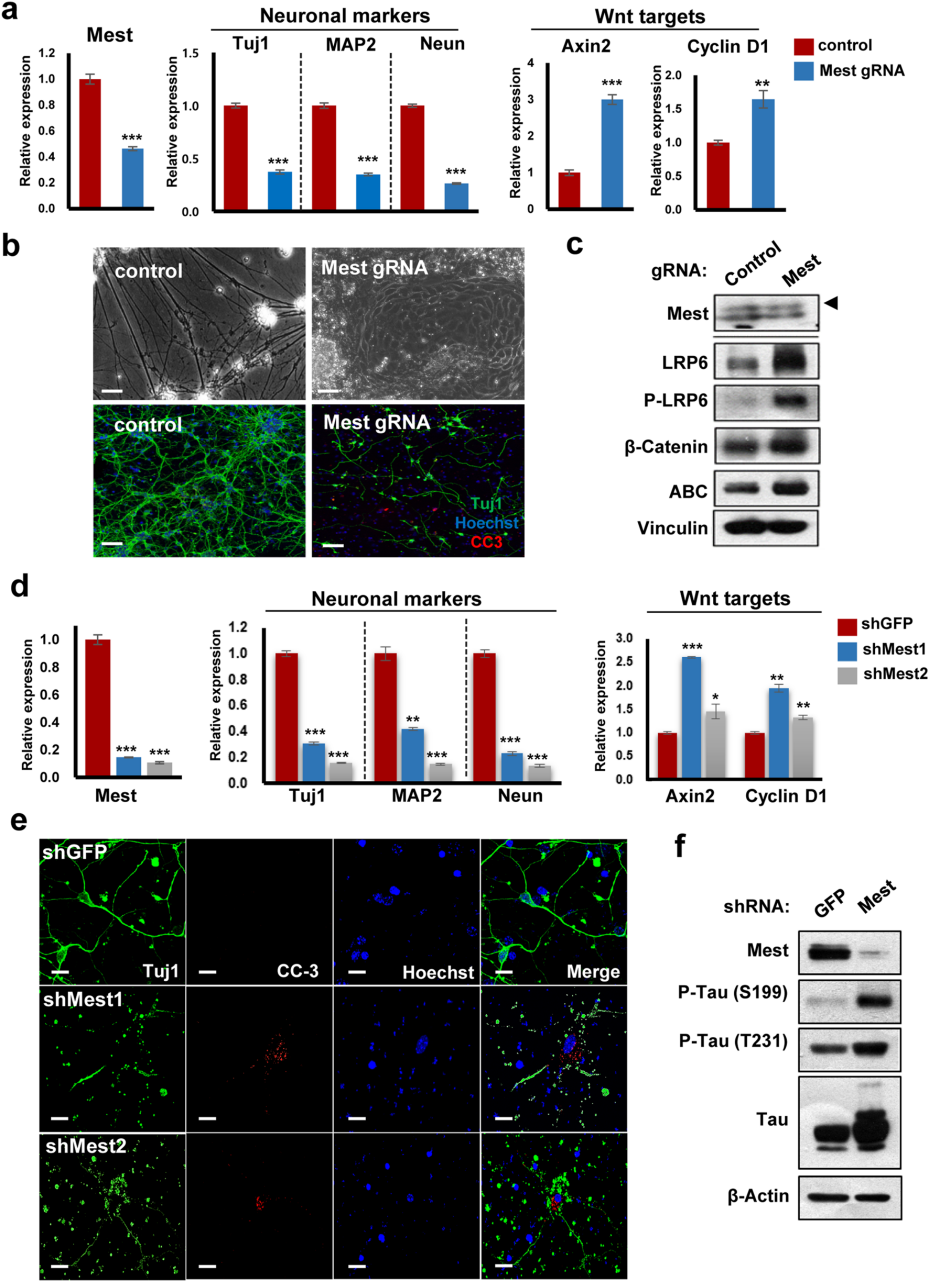
Mest depletion results in elevation of Wnt signaling and Tau phosphorylation, leading to neurodegeneration. (**a**) Relative mRNA expression of Mest, neuronal markers, and Wnt target genes in 11-day old neurons of P19 LSC-5 control and Mest gRNA stable cells treated with rapamycin. (**b**) Inducible knockout of Mest caused neurodegeneration in P19 Mest gRNA stable cells. P19 LSC-5 neurons treated with rapamycin were fixed and immunostained with anti-Tuj1 (green) and cleaved caspase-3 ((CC-3), (red)) antibodies. Phase contrast (top panels, scale bars, 100 μm) and fluorescent images (bottom panels) of 11-day neurons of P19 LSC-5 control and Mest gRNA stable cells induced with rapamycin. Scale bars, 88 μm. (**c**) Mest deficiency leads to increased Wnt signaling. Western blotting for Mest and the Wnt signaling components: LRP6, p-LRP6, β-catenin, and Active form of β catenin (ABC), in P19 Mest inducible KO cells isolated from (B) was performed. Vinculin was used as a loading control. Filled arrow indicates specific band. (**d**) qPCR analysis of Mest, neuronal markers, and Wnt target genes was performed with the same RNA samples harvested from hippocampal neurons infected with lentiviral shMest at DIV7 and incubated for 24 h. (**e**) Mest knockdown caused neurodegeneration in hippocampal neurons. DIV7 hippocampal neurons treated with lenti-shMest were immunostained with anti-Tuj1 (green) and cleaved caspase-3 ((CC-3), (red)) antibodies. Scale bars, 18 μm. (**f**) DIV7 rat hippocampal neurons treated with shMest lentivirus showed increased tau phosphorylation at S199 and Thr231 sites. All mRNA levels were normalized to that of β-actin. Data are shown as mean ± SD; *, *p* < 0.05; **, *p* < 0.01; ***, *p* < 0.001; ****, *p* < 0.0001 compared with the corresponding control group. Student’s *t* test was used for statistical analysis.

Next, to mimic the low levels of Mest in AD patients, we employed an inducible split-Cas9 system to suppress endogenous Mest expression^21^. LSC-5 Mest gRNA was constructed and transduced into P19 cells (Supplementary Fig. 4a, b). Treatment of P19 LSC-5 Mest gRNA stable cells with rapamycin suppressed Mest mRNA expression and increased Wnt target gene levels (Fig. 3a). Mest inducible KO (MiKO) cells also showed a reduction in the levels of neuronal markers. MiKO cells formed large aggregates, whereas control cells exhibited proper neurite formation (Fig. 3b). Immunofluorescence examination showed that control cells had intact neuron morphology with decent neuronal differentiation upon staining with Tuj1, whereas MiKO cells displayed fragmented neurites with few surviving neurons and positive signals for cleaved caspase 3 (Fig. 3b). Levels of Wnt signaling components such as p-LRP6, β-catenin, and active form of β-catenin (ABC) were also elevated in MiKO cells (Fig. 3c). These findings suggest that Mest suppression forced the activation of Wnt signaling, resulting in neurodegeneration.

To corroborate our findings, primary hippocampal neurons were transduced with the shMest lentivirus, and the RNA samples were isolated from the same infections to perform Real time PCR analysis for neuronal markers and Wnt target genes (Axin 2 and cyclin D1). The mRNA expression of neuronal markers in Mest knockdown cells was significantly downregulated, while the expressions of Wnt target genes were upregulated (Fig. 3d). A recent study showed increased Wnt signaling in the brains of AD patients^28^ which is consistent with our findings. Mest knockdown neurons also stained positive for cleaved caspase 3 (CC3), suggesting the occurrence of apoptosis, while shGFP-treated cells remained intact with the expression of Tuj1 (Fig. 3e). Tau phosphorylation causes the formation of neurofibrillary tangles. A gradual increase in Tau phosphorylation at Tyr18, Thr231, and Ser199 sites correlate with AD severity^29^. Interestingly, we found that Mest knockdown in rat cortical neurons increased tau phosphorylation at Ser199 and Thr231 residues (Fig. 3f). Overall, these results demonstrate that Mest suppression results in increased Wnt signaling and tau phosphorylation, possibly causing neurodegeneration.

## Discussion

Wnt signaling plays an essential role in neuron maturation, synaptogenesis, axonal remodeling, and long-term potentiation. These processes are impaired in AD, lending support to the view that Wnt signaling is a possible choice for genetic studies aimed at understanding AD pathogenesis^30–32^. The changes in protein levels were most noticeable in β-catenin, GSK3β, and transcription factor 7 like 1(TCF7L1), indicative of altered Wnt signaling in AD^33^. In this regard, common genetic variations (single nucleotide polymorphisms) of LRP6, Disheveled 1, and GSK3β are found in AD^3,34–36^. Although the mechanisms are not entirely understood, Wnt signaling has both pathogenic and protective roles in AD^37^. In rat hippocampal neurons, Wnt3a treatment showed protective function by reducing Aβ toxicity and tau phosphorylation^38^. In contrast, familial Alzheimer’s disease (FAD)-linked presenilin 1 (PSEN1) mutations (M146L and M146V) increase β-catenin levels and Wnt target gene expression, resulting in pathogenic effects^39,40^. Our data support the hypothesis that over-activation of Wnt signaling upon Mest depletion causes neurodegeneration of hippocampal neurons (Fig. 3).

AD progression may potentiate pathological changes due to epigenetic regulation. The basis for methylation changes in AD are still unclear. Studies have shown that the promoter region of the amyloid precursor protein (APP) gene is hypomethylated after the age of 70 years, which might result in its aberrant expression, thus generating excessive Aβ^41^. Neprilysin is a vital Aβ-degrading enzyme in the brain and its promoter is highly methylated, resulting in the accumulation of Aβ^42^. The current study revealed that Mest promoter hypermethylation may be the epigenetic mechanism responsible for the reduction of Mest mRNA levels in AD patients (Fig. 1). Our results also corroborate previous reports that the Mest promoter is hypermethylated and its mRNA level is significantly decreased in AD brains^18,43^. The reasons for the enrichment of hypermethylated alleles in the Mest promoter in AD patients is unclear and requires further study. It will be interesting to elucidate the upstream mechanisms that cause hypermethylation of the Mest promoter in the brains of AD patients.

Tau protein is predominantly phosphorylated at Ser/Thr-Pro sites, causing the formation of neurofibrillary tangles^44^. In the present study, we showed that Mest depletion caused tau hyperphosphorylation at the Ser199 and Thr231 sites in rat cortical neurons (Fig. 3f). Microarray studies on AD cortex with neurofibrillary tangles also showed diminished Mest levels^45^. Determining the tau kinase responsible for tau phosphorylation upon Mest reduction requires further investigation.

The current results should be interpreted with caution, as a larger number of AD patient samples should be analyzed. The role of Mest in other signaling pathways remains unclear. In conclusion, we found that hypermethylation of the Mest promoter causes a reduction in Mest mRNA levels and activation of Wnt signaling in the brain tissues of AD patients. Unfolding the epigenetic modifications i.e. Mest promoter hypermethylation, may provide new insights into the underlying mechanisms that influence neurodegeneration and AD progression.

## Supplementary Information

Supplementary Information 1.

## Data Availability

All data generated or analyzed during this study are included in this article and its supplementary information files.
