# Supplementary material for: Hypermethylation of Mest promoter causes aberrant Wnt signaling in patients with Alzheimer’s disease
Source: Sci Rep. 2021 Oct 8;11:20075. doi: 10.1038/s41598-021-99562-9 (PMC8501037; doi:10.1038/s41598-021-99562-9)

**Supplementary Information File**

**Hypermethylation of Mest promoter causes aberrant**

**Wnt signaling in patients with Alzheimer’s disease**

Renuka Prasad^1,†^, Hwajin Jung^1,†^, Anderson Tan^1^, Yonghee Song^1^, Sungho Moon^1^, Mohammed R. Shaker^2^, Woong Sun^2^, Junghee Lee^3^, Hoon Ryu^3^,^4*^, Hyun Kook Lim^5*^ and Eek-hoon Jho^1*^

^†^ These authors contributed equally to this work

^*^ To whom correspondence may be addressed.

E-mail: [hoonryu@bu.edu](mailto:hoonryu@bu.edu), [drblues@catholic.ac.kr](mailto:drblues@catholic.ac.kr), [ej70@uos.ac.kr](mailto:ej70@uos.ac.kr)

^1^ Department of Life science, University of Seoul, Seoul 02504, Republic of Korea.

^2^ Department of Anatomy, Korea University College of Medicine, Seoul 02841, Republic of Korea.

^3^ Boston University Alzheimer’s Disease Center and Department of Neurology, Boston University School of Medicine, Boston, MA 02118, USA.

^4^ Center for Neuroscience, Brain Science Institute, Korea Institute of Science and Technology, Seoul 02792, Republic of Korea.

^5^ Department of Psychiatry, Yeouido St. Mary's Hospital, College of Medicine, The Catholic University of Korea, Seoul, Korea.

**This PDF file includes:**

Supplementary Figures 1 to 4 and Supplemental Tables 1 to 4


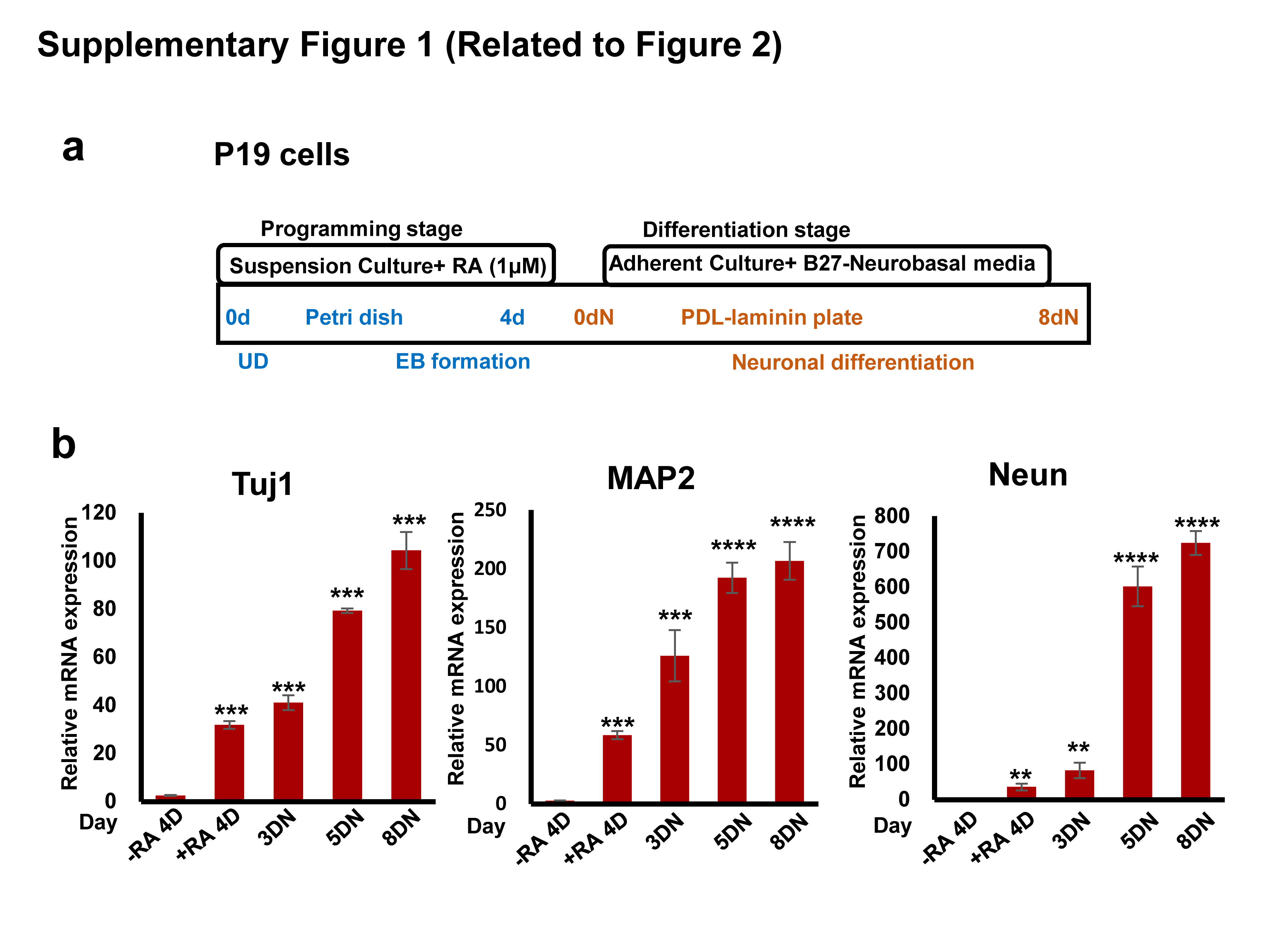
**Supplementary Figure 1. Increased expression of neuronal markers during neuronal differentiation of P19 cells.**

**a.** Scheme of P19 neuronal differentiation. UD, undifferentiated; EB, embryonic body, 8dN; 8-day neuron, RA, retinoic acid. **b.** qPCR analyses of Tuj1, MAP2, and Neun mRNA expression during neuronal differentiation. The mRNA levels were normalized to that of β-actin. Data are shown as mean ± SD; * p < .05; ** p < .01; *** p < .001; **** p < .0001 compared with the corresponding control group. Student’s t-test was used for statistical analysis.

**
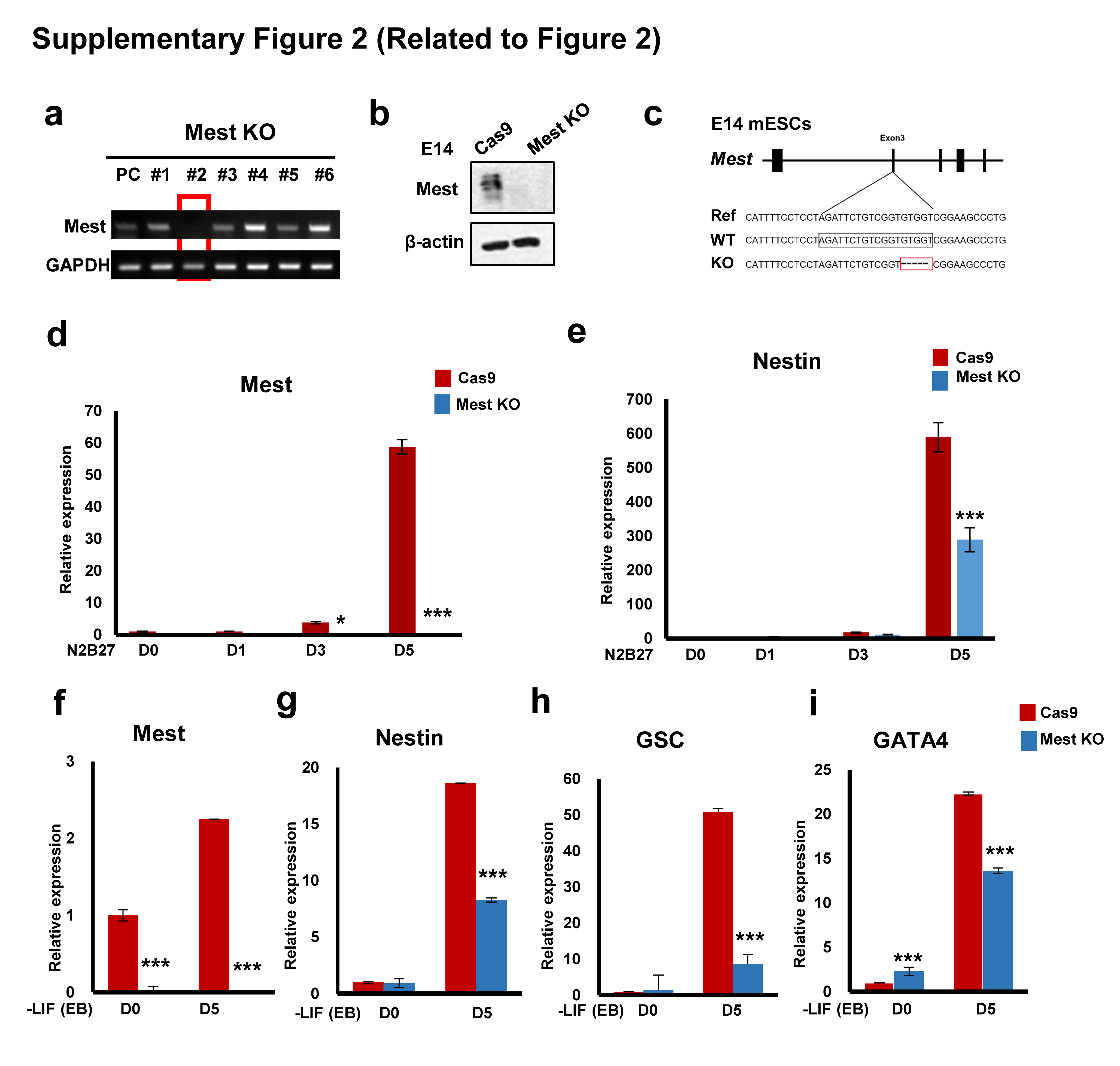
**

**Supplementary Figure 2. Mest knockout (KO) in E14 mESCs leads to delayed differentiation.**

**a.** Mest mRNA expression in E14 mESC cells transduced with Mest gRNA was analyzed using RT-PCR. Clone 2 showed absence of Mest transcripts. **b.** Mest protein levels in clone 2. Protein lysates were extracted from clone 2 and Cas 9 (control). Mest expression levels were analyzed using western blotting. **c.** Representative Sanger sequencing trace of one Mest targeted clone showing a mutation within Mest exon 3 in E14 mESCs. The black box indicates the Mest gRNA target region. **d.** Mest mRNA levels were increased in control cells, but not in Mest KO cells. qPCR analysis of Mest mRNA expression during mESC differentiation in N2B27 medium **e.** Nestin mRNA expression levels were reduced in Mest KO cells upon neuronal differentiation in N2B27 medium. **f.** Mest mRNA expression was absent in Mest KO cells, while the control cells showed increased Mest mRNA expression during embryonic body differentiation. **g-i.** mRNA expression of ectodermal (Nestin), mesodermal (GSC), and endodermal (GATA4) markers was reduced in Mest KO cells. The mRNA levels were normalized to that of β-actin. Data are shown as mean ± SD; * p < .05; ** p < .01; *** p < .001; **** p < .0001 compared with the corresponding control group. Student’s t-test was used for statistical analysis.

**
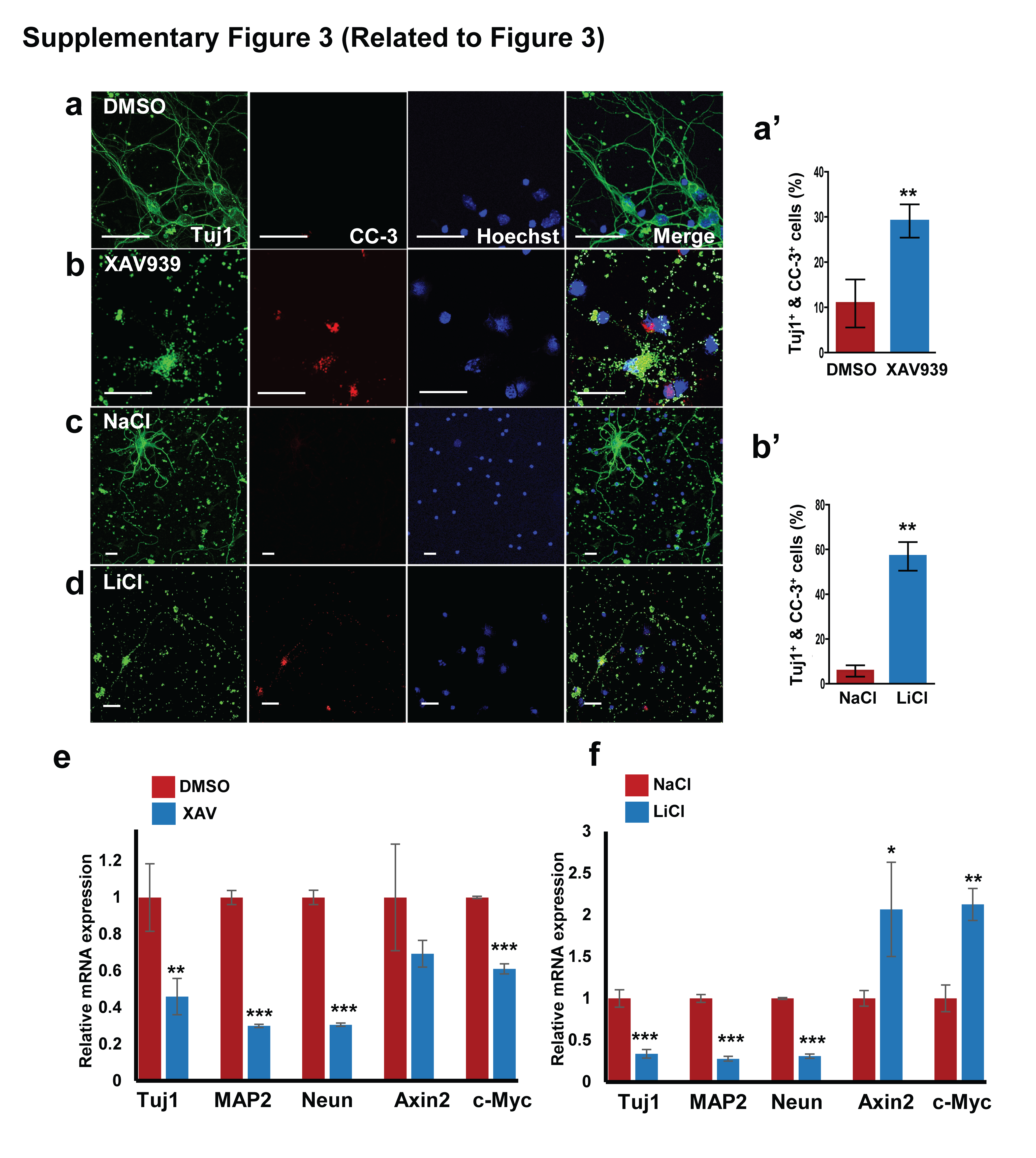
**

**Supplementary Figure 3. Either extreme of Wnt signaling causes neurodegeneration in neurons**.

**a-d:** Primary mouse hippocampal neurons cultured *in vitro* for 7 days were incubated with the small molecules indicated in the figure for 48 h and then immunostained with anti-Tuj1 (green) and cleaved Caspase-3 ((CC-3), (red)) antibodies. DMSO, 5μM XAV939; 10mM Nacl,10mM LiCl. **a-b;** To determine the percentage of Tuj1^+^ and cleaved caspase-3(CC-3)^+^ cells in each sample, the number of Tuj1^+^ & CC-3^+^cells was divided by the number of hoechst^+^ cells in four randomly selected areas. Scale bars, 25μm. For each sample, more than 150 DAPI+ cells were counted. Data are expressed as mean ±SEM from the indicated number of samples. **e-f:** The P19 8-day differentiated neurons were treated with the small molecules indicated in the figure for 48 h and then harvested for qPCR analysis. DMSO, XAV (5μM); 10mM NaCl, 10mM LiCl. The mRNA levels were normalized to that of β-actin. Data are shown as mean ± SD; * p < .05; ** p < .01; *** p < .001; **** p < .0001 compared with the corresponding control group. Student’s t-test was used for statistical analysis.

**
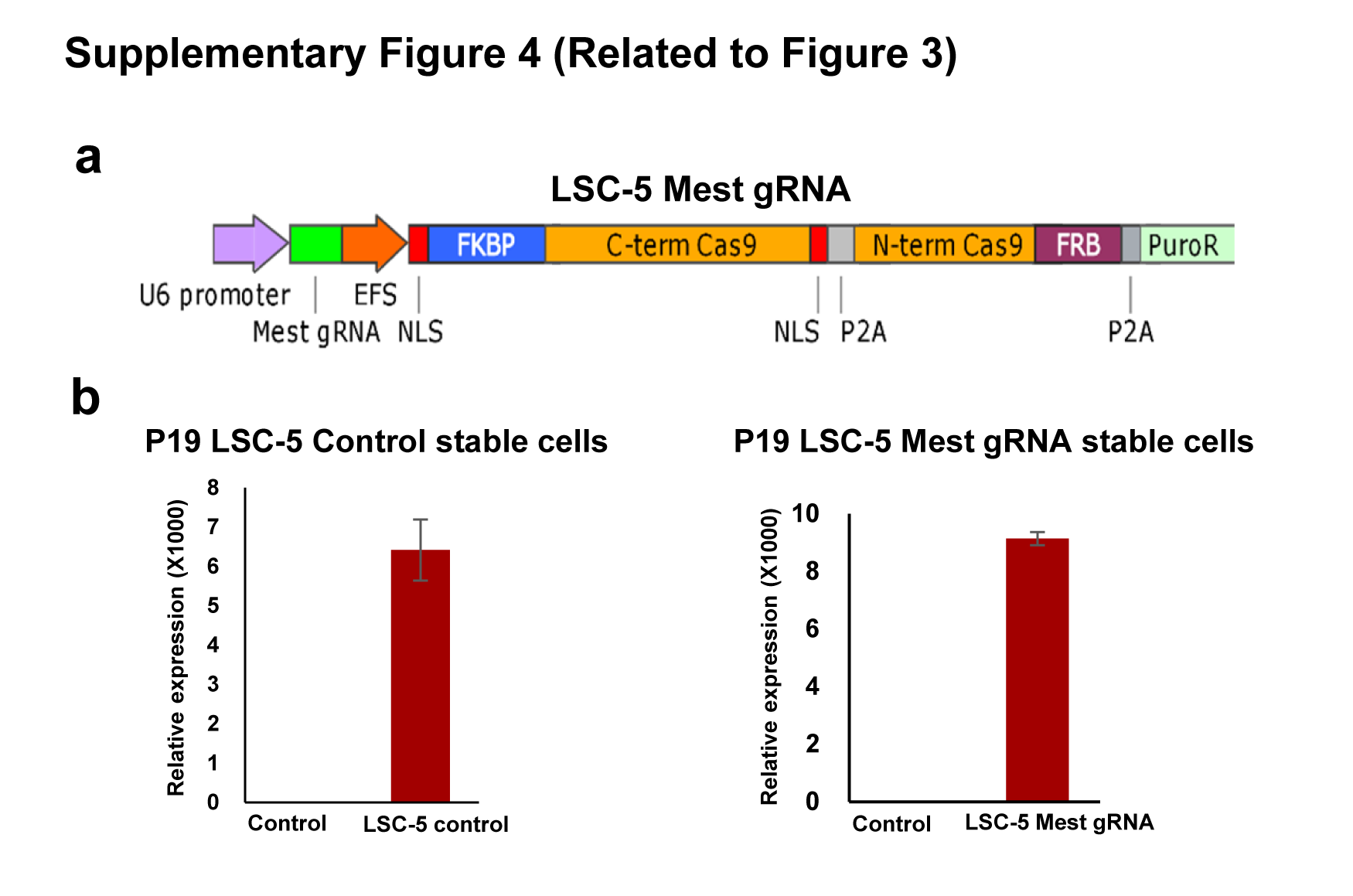
**

**Supplementary Figure 4. Generation and validation of P19 LSC-5 stable cells**

**a.** Linear map of lentiviral split-Cas9 plasmid containing U6 promoter–driven Mest gRNA. **b.** LSC-5 control and Mest-gRNA lentivirus were transduced into P19 cells and selected using puromycin. The expression of the LSC-5 vector component FRB was validated in P19 LSC-5 control and Mest gRNA stable cells using qPCR. These clones were used in further experiments.

**Supplementary Tables 1 to 4**

**Supplementary Table 1. Clinical characteristics of the non-demented control subjects in the study**

| NPID | Braak  stage | Sex | Cause of death | Age | CDR | Methylation Status |
| --- | --- | --- | --- | --- | --- | --- |
| Control 3 | 1 | F | N/A | 101 | N/A | Hyper |
| Control 4 | 1 | F | N/A | 87 | N/A | Normal |
| Control 5 | 1 | M | N/A | 88 | 0 | Normal |
| Control 105 | 2 | M | Other | 86 | N/A | Normal |
| Control 114 | 2 | F | Other | 87 | N/A | Normal |
| Control 135 | 3 | M | N/A | 89 | N/A | Normal |
| Control 140 | 1 | M | N/A | 67 | N/A | Normal |
| Control 81 | 1 | M | Other | 82 | N/A | Normal |
| Control 85 | 1 | M | N/A | 67 | N/A | Normal |

N/A: Not available, CDR: Clinical Dementia Rating

**Supplementary Table 2. Clinical characteristics of Alzheimer’s disease subjects in the study**

| NPID | Braak  stage | Sex | Cause of death | Age | CDR | Methylation Status |
| --- | --- | --- | --- | --- | --- | --- |
| AD 20 | 5 | M | AD | 82 | N/A | Hyper |
| AD 37 | 6 | F | AD | 79 | N/A | Normal |
| AD 40 | 6 | M | AD | 70 | N/A | Normal |
| AD 41 | 6 | M | AD | 59 | N/A | Normal |
| AD 132 | 5 | F | AD | 80 | N/A | Normal |
| AD 133 | 5 | M | AD | 92 | N/A | Hyper |
| AD 2 | 5 | F | AD | 90 | N/A | Hyper |
| AD 3 | 5 | M | AD | 100 | 3 | Hyper |
| AD 5 | 5 | M | AD | 75 | N/A | Normal |

**Supplementary Table 3. Primers used for qPCR and Mest promoter amplification**

| **qPCR primers** | |
| --- | --- |
| Mest-F | CTGGGAAGGGCTGACCCTGAGGTTC- |
| Mest-R | CCATTCGACAGACAGAGACTCTTTATG |
| Tuj1-F | TAGACCCCAGCGGCAACTAT |
| Tuj1-R | GTTCCAGGTTCCAAGTCCACC |
| NeuN-F | CAACATCCCCTTCCGGTTC |
| NeuN-R | TGACCTCAATTTTCCGTCCC |
| Axin2-F | ATTCGGCCACTGTTCAGACG |
| Axin2-R | GACAACCAACTCACTGGCCTG |
| β-Actin-F | GCGGGAAATCGTGCGTGACATT |
| β-Actin-R | GATGGAGTTGAAGGTAGTTTCGTG |
| GAPDH-F | CGACTTCAACAGCAACTCCCACTCTTCC |
| GAPDH-R | TGGGTGGTCCAGGGTTTCTTACTCCTT- |
| **Primers for bisulfite sequencing of Mest promoter region** | |
| Mest-F | TC/TGTTGTTGGTTAGTTTTGTAC/TGGTT |
| Mest-R | AAAAATAACACCCCCTCCTCAAAT |

**Supplementary Table 4. Primer sequences for gRNA, shRNA, and T7E1 assays**

| **gRNA sequences** | |
| --- | --- |
| gRNA-Mest-sense | gtggaaaggacgaaacaccgAAGATTCTGTCGGTGTGGTgttttagagctagaaatagc |
| gRNA-Mest-antisense | ccatttgtctcgaggtcgagCAGGAAACAGCTATGACC |
| **T7E1 primers** | |
| PCR product  (1078 base pairs)- F | TGACCCCCTCTCATGGGTTA |
| PCR product  (1078 base pairs)- R | GGAGAGCGTAACTCCAGCTT |
| PCR product  (441 base pairs)- F | GTCCTGTCAGCTTGCTTTCTAACA |
| PCR product  (441 base pairs)- R | GGAGAGCGTAACTCCAGCTT |
| **shRNA sequences** | |
| shMest1 | CACCGGCCATTGGATCCTATAAATCGAAATTTATAGGATCCAATGGCC |
| shMest2 | CACCGCCACATCAGTACTCCATATCGAAATATGGAGTACTGATGTGG |

**Raw data of Western blots**


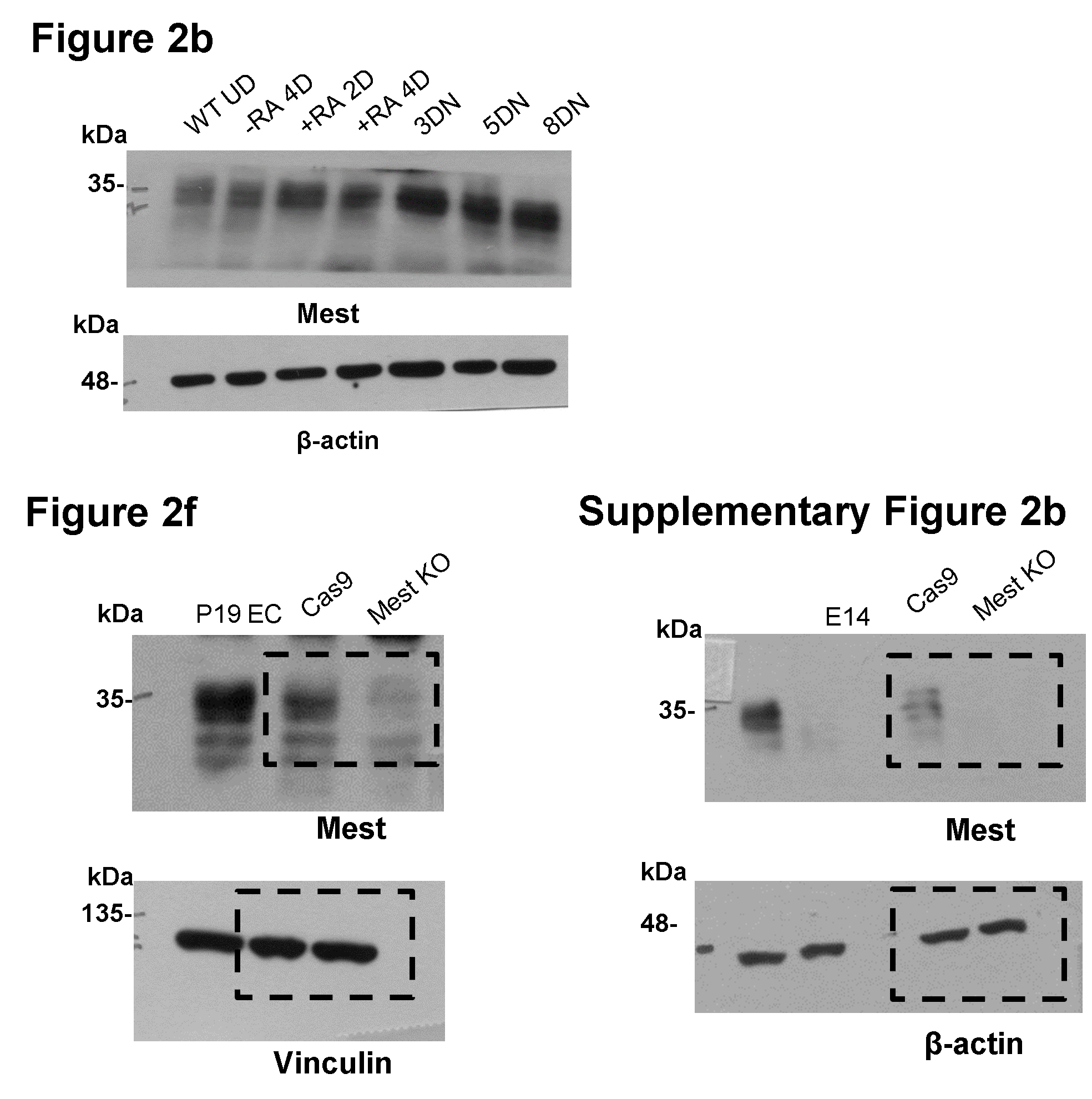


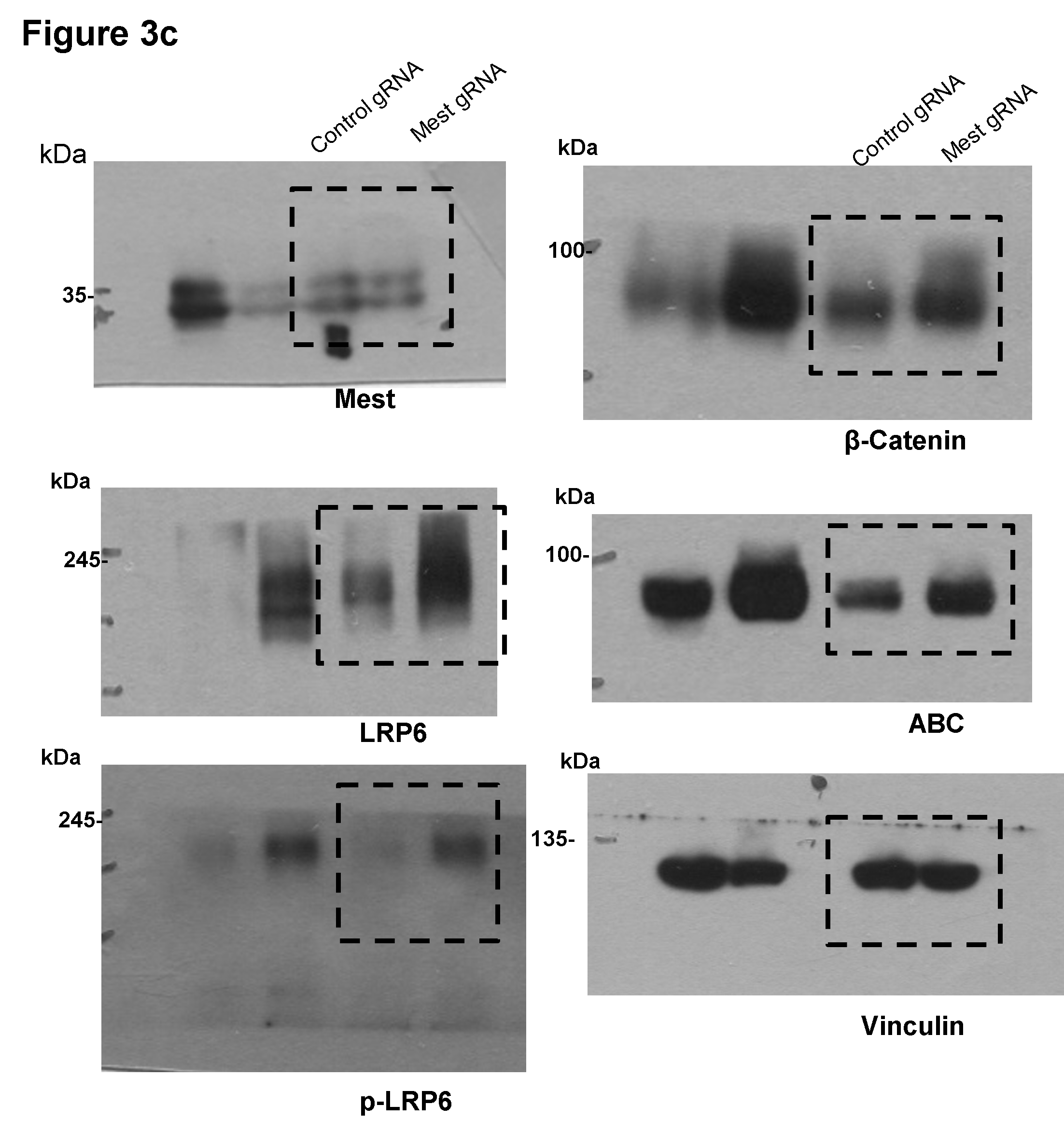


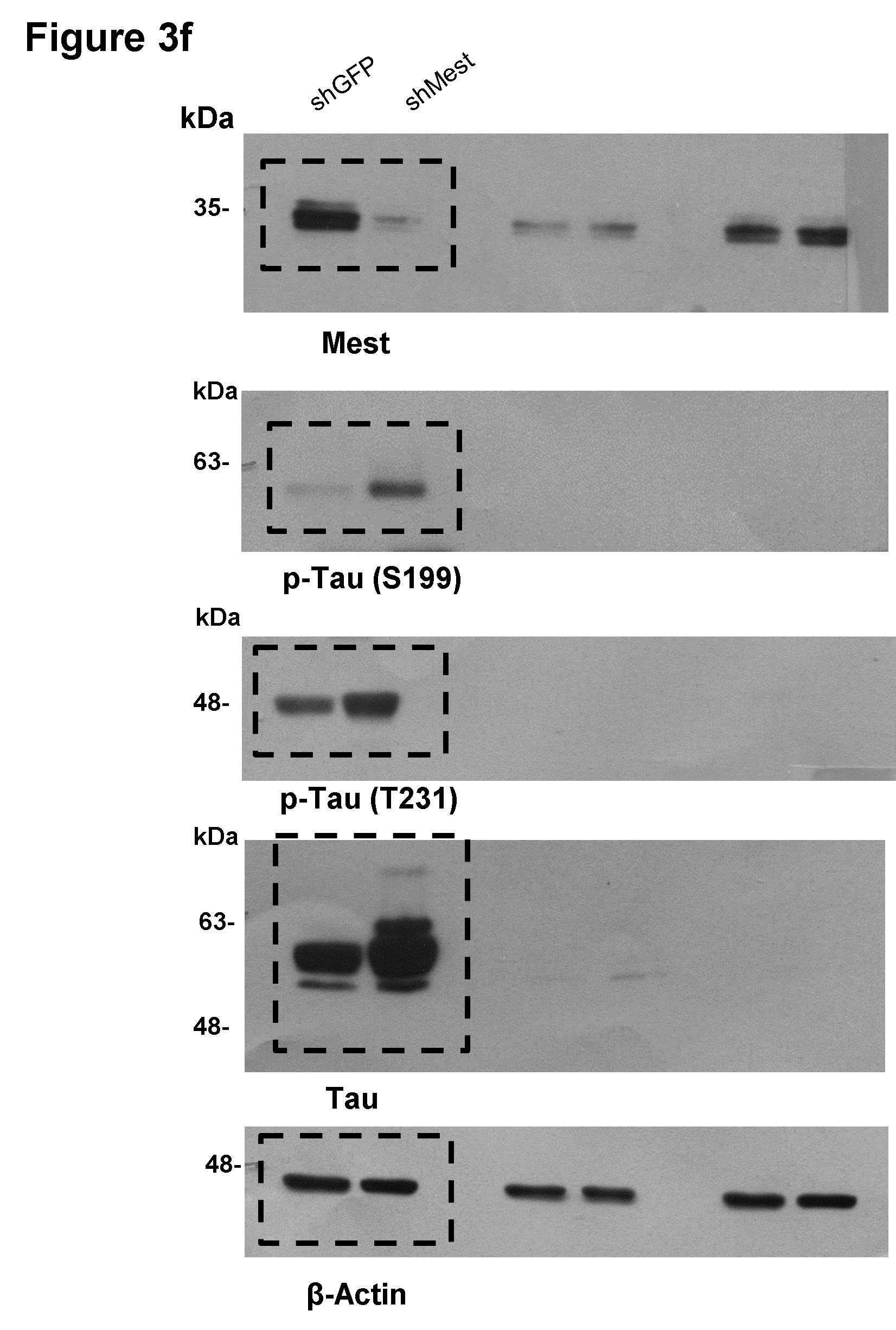

Supplement: Supplementary file 1 — Supplementary Information 1. [file 41598_2021_99562_MOESM1_ESM.docx]
